# Pneumocystis jirovecii Pneumonia (PCP) in a Non-HIV Lung Cancer Patient in the Absence of Common Risk Factors

**DOI:** 10.7759/cureus.45458

**Published:** 2023-09-18

**Authors:** Shahnawaz Hashmi, Dawood Yousuf, Prasad Kumar

**Affiliations:** 1 Internal Medicine, University Hospitals Bristol and Weston NHS Foundation Trust, Weston-Super-Mare, GBR; 2 Pulmonology, University Hospitals Bristol and Weston NHS Foundation Trust, Weston-Super-Mare, GBR

**Keywords:** radiotherapy (rt), immunocompromised, bal, pcr, squamous cell lung cancer, non-hiv patients, pcp

## Abstract

Pneumocystis jirovecii pneumonia (PCP) has been described mainly in AIDs and in immunocompromised patients with hematological malignancies, organ transplant recipients, collagen vascular disease, and primary immune deficiencies or those under treatment with steroids or chemotherapy. The incidence of PCP pneumonia is increasing in solid organ tumors and hematological malignancies receiving chemotherapy. Pneumocystis pneumonia has been rarely reported in patients with non-small cell lung cancer (NSCLC).

We describe a 68-year-old woman with a recent diagnosis of squamous cell lung cancer, who received radiotherapy two weeks prior to the current hospital admission with shortness of breath and dry cough. The initial investigations, including chest X-ray and CT images, were suggestive of atypical pneumonia, with PCP pneumonia as the top differential. Treatment was started with high-dose trimethoprim-sulfamethoxazole (cotrimoxazole) and oxygen support. Serum beta-glucan was found to be more than 500 pg/ml in favor of PCP infection. Oral steroids were added to the treatment in view of hypoxia (arterial oxygen pressure (PaO2) < 70 mmHg) requiring high-flow nasal cannula support. Subsequently, bronchoscopy was done and the bronchoalveolar lavage (BAL) sample came positive for PCP polymerase chain reaction (PCR). The patient made a significant recovery after four weeks of treatment with cotrimoxazole and was discharged home in stable condition with cotrimoxazole prophylaxis.

The reported cases of PCP pneumonia in lung cancers were following chemotherapy, chemoradiation, or steroid treatment. The incidence of PCP pneumonia in lung cancer patients receiving radiotherapy is relatively rare. Our patient could not tolerate chemotherapy for the cancer due to an anaphylactic reaction and hence was treated with radiotherapy alone for the lung cancer prior to getting PCP pneumonia. Therefore, it is important to carry a high index of suspicion for PCP infection in a lung cancer patient presenting with features of atypical pneumonia following cancer treatments, including radiotherapy alone.

## Introduction

Pneumocystis pneumonia (PCP) is a serious infection caused by the fungus Pneumocystis jirovecii. Antonio Carinii isolated this organism for the first time in rat lungs in 1910. It was thought to be a trypanosome because of its two distinct life cycles and response to pentamidine. Hence, it was named Pneumocystis carinii owing to its unique tropism for the lungs. Later on, the classification was changed to fungi, and the name was changed to Pneumocystis jirovecii after the pathologist Jirovec, who first discovered it in humans [1]. PCP is an opportunistic infection that causes significant morbidity and mortality in the immune-compromised population. This population has been growing and diversifying, yet contemporary epidemiology is lacking in the UK. The incidence of PCP increased from 2.2 to 4.5 per 100,000 population, between 2012/2013 and 2019/2020, as was observed in a large study conducted by Katharine Pates et al. [2]. PCP is characterized by symptoms like dry cough, dyspnoea, low-grade fever, tachypnoea, and hypoxemia. PCP spreads through the air, from person to person. Some healthy adults can carry the Pneumocystis fungus in their lungs without having symptoms, and it can spread to other people, including those with weakened immune systems [3]. Most people who get PCP have compromised immune systems. About 30-40% of people who get PCP have HIV [4]. The other people who are prone to getting PCP are usually taking corticosteroids that lower the body’s ability to fight germs or sickness or have other medical conditions, such as chronic lung diseases, inflammatory diseases, autoimmune diseases (for example, lupus or rheumatoid arthritis), and solid organ or hematopoietic cell transplant [5]. In this case, we will describe a scenario of a patient who developed PCP pneumonia in the absence of these commonly associated risk factors.

## Case presentation

We report this case of a 68-year-old, non-diabetic, ex-smoker female who presented to the Accident & Emergency department of Weston General Hospital with complaints of shortness of breath (SOB) at rest and dry cough for six days. She was tachypnoeic and tachycardic at presentation and had to be put on 4L of oxygen to maintain a saturation of 94%. Auscultation revealed wheeze bilaterally; no other specific examination finding of note was seen. An important thing of note in her medical history was a recent diagnosis (12 weeks) of squamous cell carcinoma (SCC) of the lung (T3N1M0). She received palliative radiotherapy for it, the last round of which was two weeks prior to coming to the hospital. She could not complete chemotherapy (paclitaxel), as she developed anaphylaxis on the very first dose. She had no other significant past medical history. Initial investigations were done, including blood testing, electrocardiogram, chest X-ray, atypical screen (legionella, pneumococcal antigens), and respiratory swabs. Her investigations are shown below in Tables 1, 2.

**Table 1:** Initial bloods FBC - full blood count, RBC - red blood count, MCV - mean corpuscular volume, MCH - mean corpuscular hemoglobin, MCHC - mean corpuscular haemoglobin concentration, RDW - red cell distribution width, INR - international normalized ratio, aPTT - activated partial thromboplastin time

FULL BLOOD COUNT	RESULT	UNIT	REFERENCE RANGE
White Cell Count	11.12	10^9/L	4.0-11.0
RBC	2.76	10^12/L	3.8-5.3
Hemoglobin	78	g/L	120 - 150
Hematocrit	0.24	L/L	0.37-0.45
MCV	87.3	fL	83-100
MCH	28.3	pg	27.0 - 32.0
MCHC	324	g/L	310 - 350
Platelets	383	10^9/L	150 - 400
RDW	16.3		11.5 - 15.5
Neutrophils	10.10	10^9/L	1.5-8.0
Lymphocytes	0.26	10^9/L	1.0-4.0
Monocytes	0.69	10^9/L	0.2- 1.0
Eosinophils	0.04	10^9/L	0.0 - 0.5
Basophils	0.03	10^9/L	0.0- 0.2
FBC Comment	Sample checked. no clot found		
BLOOD FILM REPORT			
Neutrophilia with left shift and toxic changes			
Rouleaux present			
CLOTTING SCREEN			
Prothrombin time	12.4	seconds	9.5-12.0
INR	1		
APTT	28.2	seconds	23.0 - 32.0
APTT ratio	1.0		
Fibrinogen	>6.0	g/L	1.5-4.0

**Table 2:** Initial bloods ALP - alkaline phosphatase, ALT - alanine transaminase, eGFR - estimated glomerular filtration rate

TEST	RESULT	UNIT	REFERENCE RANGE
C-REACTIVE PROTEIN			
CRP	227	mg/L	<6.0
MAGNESIUM			
Magnesium	0.63	mmol/L	0.7 - 1
PHOSPHATE			
Phosphate	0.83	mmol/L	0.8-1.5
CALCIUM GROUP			
Albumin	16	g/L	35- 50
Calcium	2.06	mmol/L	2.2- 2.6
Adjusted Calcium	2	mmol/L	2.20 - 2.60
UREA, CREATININE, AND ELECTROLYTES			
Sodium	132	mmol/L	133 - 146
Potassium	4.0	mmol/L	3.5 - 5.3
Urea	3.8	mmol/L	2.5-7.8
Creatinine	43	umol/L	45 - 84
eGFR	>90	mL/min	eGFR >90 Normal
LIVER FUNCTION TESTS			
Total Bilirubin	3	umol/L	<21
ALP	95	U/L	30 - 130
ALT	8	U/L	10 - 50
Total Protein	50.00	g/L	60-80
Globulin	34	g/L	22 - 36

**Figure 1:**
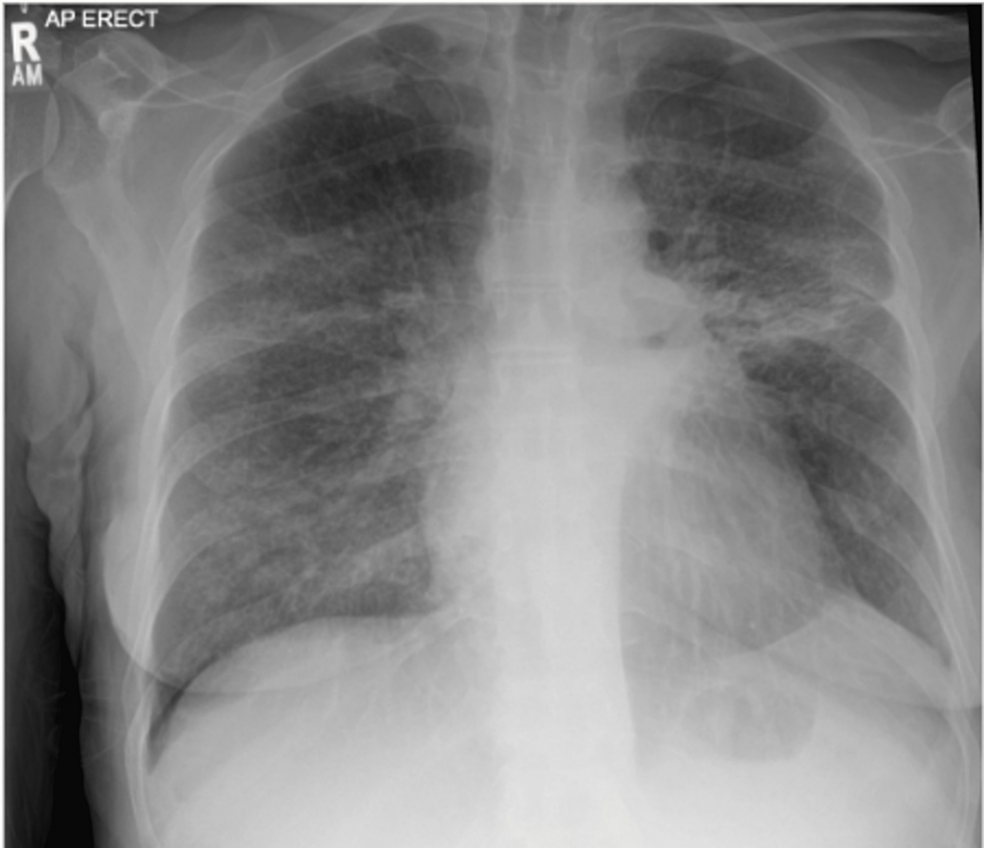
CXR: Note the bilateral, diffuse, fine reticular opacities seen in PCP pneumonia PCP: Pneumocystis jirovecii pneumonia

Our patient had a chest X-ray done as part of her initial investigations, which revealed bilateral increased interstitial markings mainly in the mid zones (Figure 1). Arterial blood gas (ABG) was done at 4L of oxygen, which revealed respiratory alkalosis with a pH of 7.48, arterial pressure of carbon dioxide (PCO2) of 4.1 kPa, and arterial partial pressure of oxygen (pO2) of 10.7 kPa. It also revealed a lactate of 2.4. Bicarbonate (HCO3) was normal, with a value of 22.9 mmol/L.

Respiratory viral swabs were negative, as well as the urine legionella and pneumococcal antigens. She was initially treated as a lower respiratory tract infection with antibiotics (amoxicillin/clavulanic acid and doxycycline) and nebulizers as required for her wheeze. She also received two units of blood transfusion for her low hemoglobin.

**Figure 2:**
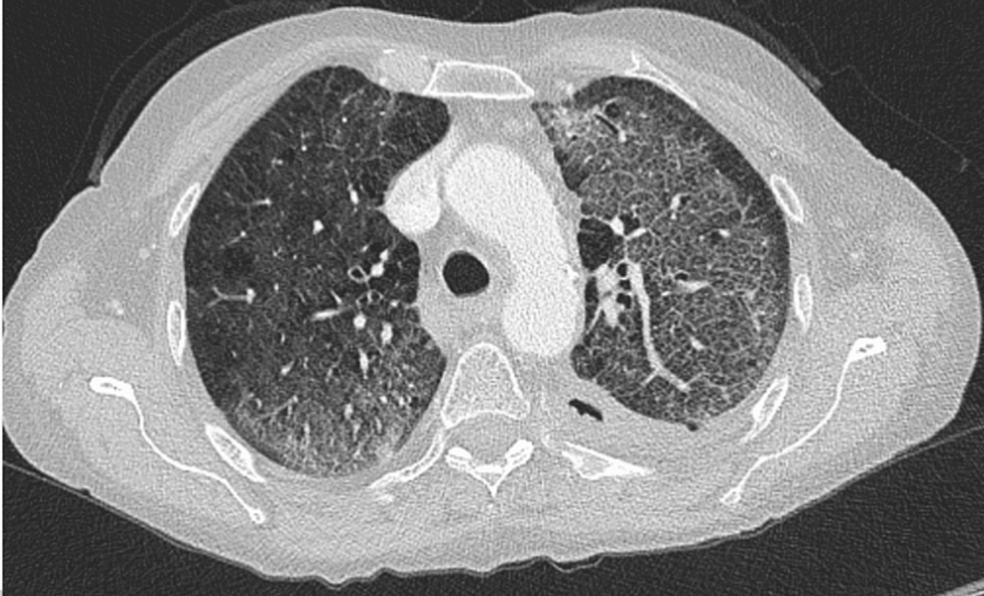
Note the GGOs with septal thickening and crazy pavement pattern. There is relative sparing of the periphery, which is one of the radiological features of PCP pneumonia. GGO: ground-glass opacity

She continued to deteriorate clinically in the next 24- 48 hours, with an increase in oxygen demand and tachypnoea present. There was no improvement seen in her lab work after the first two days of IV antibiotics. CT chest (Figure 2) with contrast was requested, which revealed diffuse ground-glass changes. There was also thickening of interlobular septum, noted in both lungs with relative peripheral lungs sparing and emphysematous changes seen. The radiologist reported that the appearance could be secondary to radiotherapy-induced pneumonitis; however, atypical infection should be considered as a strong differential diagnosis. Subsequently, beta D-glucan and Aspergillus antigen testing was done. Aspergillus antigen came back as negative, however, beta D-glucan was elevated (>500 pg/ml).

**Figure 3:**
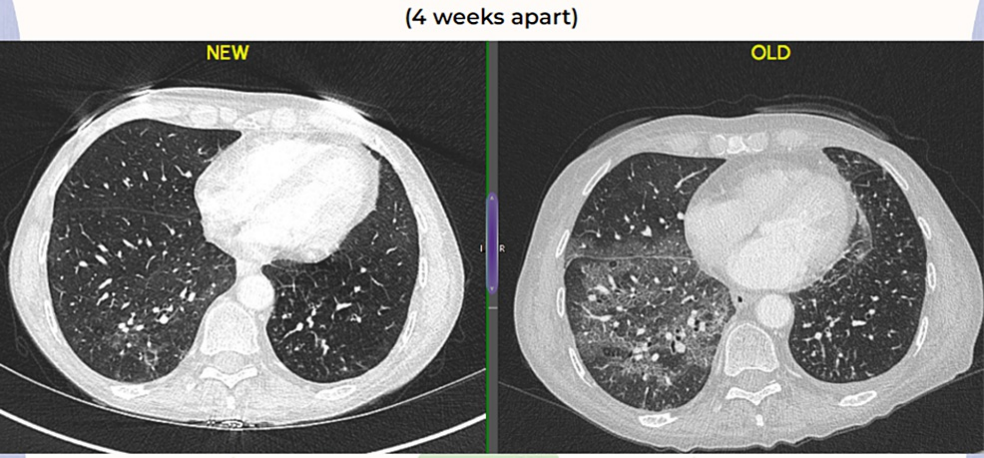
Comparative CT chests four weeks apart

Given the clinical picture, CT findings, and positive beta D-glucan test, bronchoscopy with bronchoalveolar lavage was performed. BAL samples were sent for routine microscopy and culture, fungal culture, and PCP screen. A sample for Pneumocystis jiroveci PCR was sent to the United Kingdom Health Security Agency (UKHSA) center in Manchester, where PCP was confirmed. The rest of the BAL cultures came back negative. As PCP is an AIDS-defining disease, HIV testing was done which came back negative. She was initially started on IV cotrimoxazole 1680 mg four times a day for 11 days then switched to oral. Adjunctive treatment was started with prednisolone 40 mg OD, as the patient continued to require oxygen support. The patient deteriorated slightly after the oral switch with an increase in O2 demand and worsening C-reactive protein (CRP) and WCC. A microbiologist was consulted, who advised to switch her back to IV after two days. She received a total of 21 days of co-trimoxazole treatment dose and then was continued on a prophylactic dose of 960 mg twice a day. She started improving clinically and was weaned off oxygen. Prednisolone was slowly weaned off once she was off oxygen. Her repeat CT scan done after four weeks of treatment is shown in Figure 3 as compared with the initial scans. The repeat scan showed significant improvement in the ground glass opacities and interstitial changes. She stayed for a total of six weeks in the hospital and was then discharged home with 480 mg co-trimoxazole twice a day as was recommended by the clinical microbiologist, with a follow-up arranged in the clinic. She remains under the fungal multidisciplinary team (MDT) clinic as of now.

## Discussion

Although Pneumocystis is now categorized as a fungus, it does not respond to antifungal treatment, and neither can it be cultured in a lab. Due to the symptoms being extremely varied and vague, diagnosing or even suspecting PCP in a patient who is not “immunocompromised” per se is difficult. As PCP does not present specific clinical signs different from other typical chest infections [6], it makes it harder for clinicians to suspect it early on at presentation.

In PCP, an X-ray can even present with normal findings. A doubtful CT image in a patient not responding to conventional empiric antibiotics can be a crucial hint for suspecting PCP pneumonia and initiating further investigations for the same. Most modern-day hospitals in the developed world have access to onsite stat CT imagery. A high-resolution CT can show diffuse GGOs with or without cyst formation, central and diffuse distribution with relative subpleural sparing, reticular opacities, septal thickening, and crazy paving pattern. It can also show atypical imaging manifestations, such as masses and nodular opacities, which represent a granulomatous response to the PCP infection, known as granulomatous PJP (GPJP). HRCT has 89% specificity, 100% sensitivity, and 90% accuracy in diagnosing PCP [7]. This makes it the first investigation of choice in a suspected PCP case. Often, bronchoscopies are delayed due to logistics or other reasons, an in-time HRCT can prove life-saving.

In our case, the patient suffered from squamous cell lung cancer. As per Truong J et al., HIV remains a significant risk factor [8]. In non-HIV cases, patients with hematological malignancies and recent organ or stem cell transplant patients receiving immunosuppressants are at higher risk of developing PCP infection. Non-hematological malignancies remain a lower risk factor. In a study by Fillatre P et al., solid organ tumors were at a very low risk for PCP infections with only <25 cases per 100,000 patients/ year [9]. However, an early suspicion of an atypical infection proved pivotal in our patient’s response to treatment. With no response to regular antibiotics, a CT chest raising suspicion of PCP was the cue to start the patient on PCP-specific treatment. The arterial blood gas also clearly indicated a VQ mismatch in the alveolar-arterial gradient, which is essentially the locus for PCP pathophysiology. A bronchoalveolar lavage (BAL) done later helped collect a sample for PCP PCR and confirm the diagnosis. Hence, a high-resolution CT scan (HRCT) followed by a BAL remains the diagnostic goal standard for diagnosing PCP in such patients.

Although this infection is rare, there have been cases reported in the literature. Tae-woo Kim et al. reported a similar case of PCP pneumonia in a non-immunocompromised lung cancer patient [10]. The only difference was that the patient in that case had received surgery for the cancer, rather than radiotherapy as in our case. Ray A et al. reported a case of PCP infection in a metastatic breast cancer patient, though the patient was on biological therapy [11]. Additionally, there have been cases reported of PCP infections in malignancies but usually, patients are on chemotherapy or biological therapies [6,12]. PCP infection remains a rare occurrence either way, especially in patients suffering from lung cancer [6]. Early suspicion and investigation, even in lower-risk groups, are therefore key to a good prognosis.

## Conclusions

In conclusion, PCP infection remains a rare disease in the absence of commonly associated risk factors. As this infection is termed an AIDS-defining illness, its presence in non-HIV patients remains a rare occurrence. Our patient had squamous cell cancer and had no other risk factors that are usually associated with PCP infection. The reported cases of PCP pneumonia in lung cancers were following chemotherapy, chemoradiation, or steroid treatment. The incidence of PCP pneumonia in lung cancer patients receiving radiotherapy is relatively rare. Additionally, she had received only a single round of radiotherapy. She could not receive chemotherapy, as she developed anaphylaxis to it. Considering all of this, this was a novel and rare presentation of a PCP infection that required a high clinical suspicion for diagnosis. 
